# Bacteria-induced mineral precipitation: a mechanistic review

**DOI:** 10.1099/mic.0.001049

**Published:** 2021-04-21

**Authors:** Timothy D. Hoffmann, Bianca J. Reeksting, Susanne Gebhard

**Affiliations:** ^1^​ Department of Biology and Biochemistry, Milner Centre for Evolution, University of Bath, Claverton Down, Bath, BA2 7AY, UK

**Keywords:** biomineralization, organomineralization, biologically induced mineralization, nucleation, biogenic

## Abstract

Micro-organisms contribute to Earth’s mineral deposits through a process known as bacteria-induced mineral precipitation (BIMP). It is a complex phenomenon that can occur as a result of a variety of physiological activities that influence the supersaturation state and nucleation catalysis of mineral precipitation in the environment. There is a good understanding of BIMP induced by bacterial metabolism through the control of metal redox states and enzyme-mediated reactions such as ureolysis. However, other forms of BIMP often cannot be attributed to a single pathway but rather appear to be a passive result of bacterial activity, where minerals form as a result of metabolic by-products and surface interactions within the surrounding environment. BIMP from such processes has formed the basis of many new innovative biotechnologies, such as soil consolidation, heavy metal remediation, restoration of historic buildings and even self-healing concrete. However, these applications to date have primarily incorporated BIMP-capable bacteria sampled from the environment, while detailed investigations of the underpinning mechanisms have been lagging behind. This review covers our current mechanistic understanding of bacterial activities that indirectly influence BIMP and highlights the complexity and connectivity between the different cellular and metabolic processes involved. Ultimately, detailed insights will facilitate the rational design of application-specific BIMP technologies and deepen our understanding of how bacteria are shaping our world.

## Introduction

Bacterial activity is evident in our landscapes and throughout the geological record, where it has helped shape Earth’s mineral deposits [1]. This has occurred, to some degree, via a process known as bacteria-induced mineral precipitation (BIMP). The variety of mineral deposits that are formed through bacterial activity can take on the form of stalactites and stalagmites [2], microbialites, stromatolites and thrombolites [3, 4] as well as large-scale sedimentation [4]. More recently, the ability of bacteria to induce mineral formation has gained attention for biotechnological application. In particular, the precipitation of calcium carbonate in the form of calcite, the mineral that forms limestone, has been exploited in innovative technologies in civil engineering. The first patented application is considered to have been by Adolphe and colleagues in 1990 for biological treatment of degrading stone surfaces [5]. Since then, more technologies have been developed, with a lot of attention surrounding the concept of self-healing concrete [6–8]. Other applications of BIMP include soil consolidation or heavy metal bioremediation, and excellent recent reviews exist that cover the spectrum of such technologies in detail [9–14].

For the purposes of this review, BIMP is defined as a process by which bacterial activity indirectly induces mineral formation via the release of metabolic by-products and surface interactions with ions in the open environment [15–17]. This is in contrast to bacteria-controlled biomineralization, e.g. the formation of magnetite by magnetotactic bacteria, which is metabolically and genetically controlled by the bacteria and occurs in defined locations, e.g. magnetosomes [18–20]. The latter has been reviewed in detail elsewhere [15, 21] and will not be covered here. The minerals formed by BIMP generally have no specific function (aside from some potential ecological benefits) and can be considered an unintended and uncontrolled consequence of bacterial activity [22, 23]. Depending on the author, indirect biomineralization is sometimes subdivided further into more nuanced ‘bacteria-induced’ versus ‘bacteria-influenced’ mineral precipitation [13, 20, 24]. The boundaries between the two are, however, not clear cut and in this review no such division is made.

### Bacteria-induced mineral precipitation

Precipitation of mineral species in an aqueous system occurs when the ion concentration exceeds solubility and reaches a degree of super-saturation. Once the activation energy barrier is overcome, initial crystal nucleation occurs, in which metastable critical nuclei form that may dissolve back into the bulk phase. Subsequent aggregation of individual nuclei describes the process of crystal growth and precipitation [25–27]. Nucleation can take place either homogeneously, whereby nucleation occurs when critical nuclei form in the absence of foreign particles (via random collisions of ions or atoms in solution), or heterogeneously, whereby nucleation takes place when critical nuclei form on surfaces of foreign particles [25–27]. Such particles lower the activation energy by providing templates with spacing that enhances nucleation and thus, precipitation [25–27]. Furthermore, during the nucleation process foreign particles may aggregate, leading to the formation of mixed precipitates [28].

In BIMP, bacteria can induce biomineralization by modulating precipitation-relevant parameters like local ion concentrations or pH in the environment and/or by bacterial cells themselves providing nucleation sites for crystal formation. In general, this bacterial process involves the attraction of cations to negative charges on the cell surfaces, while metabolic activity provides the appropriate microenvironment and counter-anions so that these cations may precipitate as minerals [29]. The BIMP trait is common amongst bacteria across environments [9, 30–33], and, depending on bacterial species and environment, it can lead to a range of precipitated minerals (Table 1). The bacteria-induced formation of some of these minerals can further lead to co-precipitation of additional divalent metal cations and anions [34–36]. Indirect bacterial influence on precipitation parameters of saturation state and nucleation catalysis can be broadly separated into two contributing areas: cell surface and metabolic activity, and our current understanding of the mechanisms of these will be reviewed here.

**Table 1.** Minerals precipitated in association with bacterial activity*

Mineral	Chemical formula	Reference
**Carbonates**		
Calcite	CaCO_3_	[30]
Dolomite	CaMg(CO_3_)_2_	[111, 112]
Kutnahorite	CaMn(CO_3_)_2_	[113]
Siderite	FeCO_3_	[114]
Magnesite	MgCO_3_	[54, 115]
Otavite	CdCO_3_	[116]
Strontianite	SrCO_3_	[72]
Rhodochrosite	MnCO_3_	[117]
Cerussite	PbCO_3_	[118]
Hydrozincite	Zn_5_(CO_3_)_2_(OH)_6_	[36, 119]
Dypingite	Mg_5_(CO_3_)(OH)_2_·5H_2_O	[120]
Witherite	BaCO_3_	[121]
**Phosphates**		
Tricalcium phosphate	Ca_3_(PO_4_)_2_	[78]
Struvite	NH_4_MgPO_4_∙6H_2_O	[74, 113]
Bobierrite	Mg_3_(PO_4_)_2_·8H_2_O	[74, 122]
Baricite	(MgFe)_3_(PO_4_)_2_·8H_2_O	[74]
Vivianite	Fe_3_(PO_4_)·2H_2_O	[114]
Autunite	Ca(UO_2_)_2_(PO_4_)_2_∙10-12H_2_O	[44]
Uramphite	NH_4_UO_2_PO_4_	[101, 123]
Apatite	Ca_10_(PO_4_)_6_(OH)_2_	[124]
Pb-hydroxyapatite	Ca_2.5_Pb_7.5_(OH)_2_(PO_4_)_6_	[125]
Strengite	FePO_4_·2H_2_O	[126, 127]
Variscite	AlPO_4_·2H_2_O	[97]
**Silicates**		
Gehlenite	Ca_2_Al(AlSiO_7_)	[128]
Silica	SiO_2_	[129]
Nontronite	Na_0.3_Fe^3+^_2_(Si,Al)_4_O_10_(OH)_2_·nH_2_O	[130]
Chamosite	(Fe_5_Al)(Si_3_Al)_10_(OH)_8_	[126]
Kaolinite	Al_4_(Si_4_O_10_)(OH)_4_	[126]
**Sulphides**		
Mackinawite	FeS	[76]
Greigite	Fe_3_S_4_	[76, 131]
Pyrite	FeS_2_	[132]
Covellite	CuS	[133, 134]
Sphalerite	ZnS	[135]
Galena	PbS	[134]
Digenite	Cu_9_S_5_	[136]
**Sulphates**		
Gypsum	CaSO_4_·2H_2_O	[41, 54]
Celestite	SrSO_4_	[72]
Barite	BaSO_4_	[121, 137]
**Oxides**		
Magnetite	Fe_3_O_4_	[114]
Hematite	Fe_2_O_3_	[23, 138]
Ferrihydrite	Fe_2_O_3_·0.5H_2_O	[138]
Geothite	α-FeO(OH)	[138]
Manganite	MnOOH	[139]
Vernadite	MnO_2_	[57, 140, 141]
Hausmannite	Mn_3_O_4_	[142]
Todorokite	(Ca,Na,K)_x_(Mn^4+^,Mn^3+^)_6_O_10_·3.5H_2_O	[138]
Birnessite	(Na,Ca,K)_x_(Mn^4+^,Mn^3+^)_2_O_4_·1.5H_2_O	[138]
Uraninite	UO_2_	[143–145]
Calcium Arsenate	CaHAsO_3_	[146]

*Note that while these minerals have all been reported to be formed in association with bacterial activity, the mechanisms for their formation are not always known, and some minerals can be formed by multiple different mechanisms. The minerals listed and accompanying sources are non-exhaustive of the examples available in the literature.

### Cell surface: nucleation catalysis, saturation state and nucleation template

**Fig. 1.**
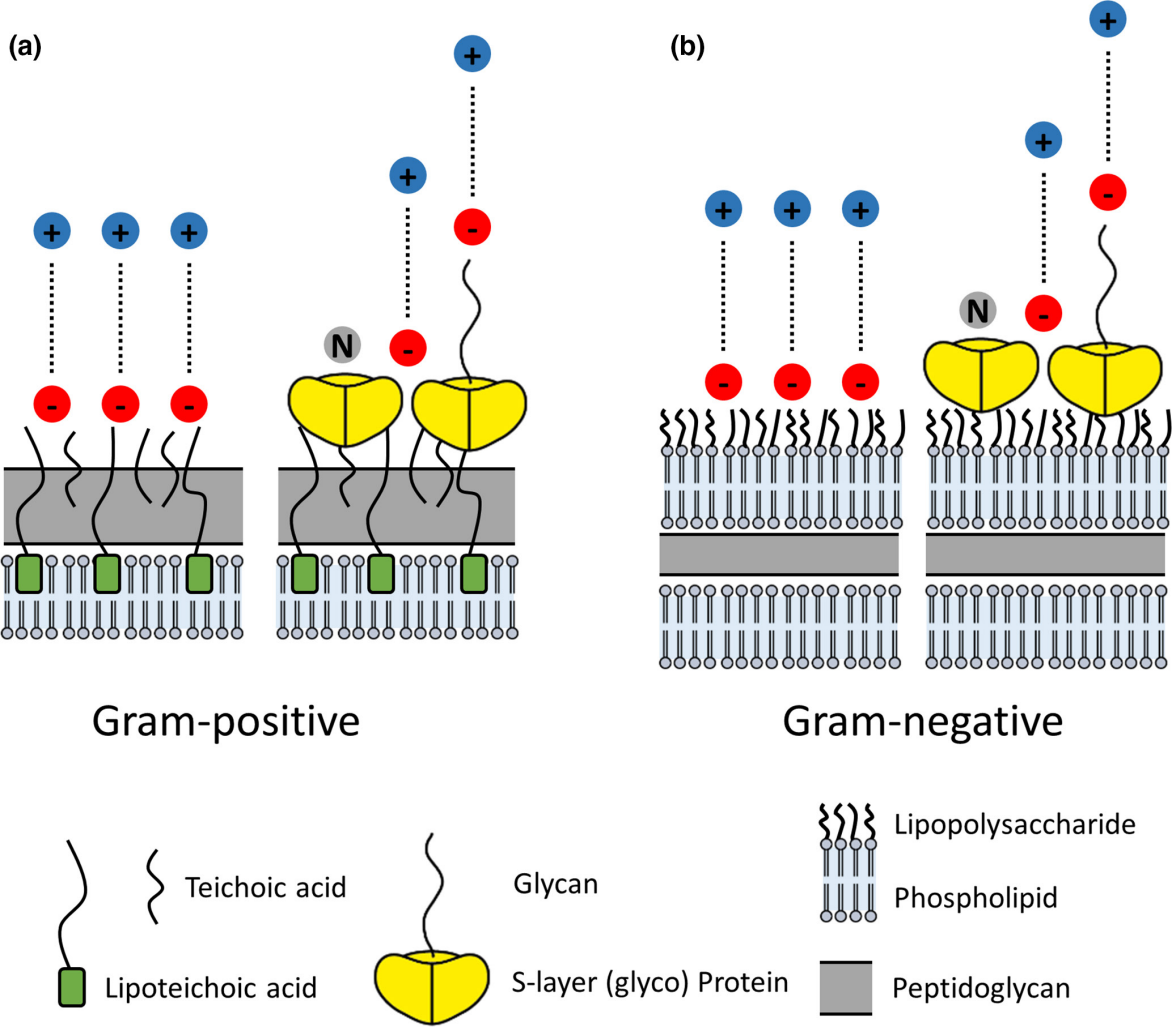
Schematic of the major supramolecular structures on the surface architecture of (a) Gram-positive and (b) Gram-negative bacteria, which provide sites for metal cation interaction. The red circles represent sites of negative charge, the grey circle represent sites of neutral charge, the blue circles represent positively charged cations, and dotted lines illustrate the attraction between negative and positive charges. Adapted from [42, 147].

The large surface area to volume ratio of bacteria make them ideal crystal nucleation sites. Covered by functional groups with a net negative charge, their surface acts as a metal cation scavenger concentrating dilute cations attracted from the environment [29, 37, 38]. Net negative surface charge is imparted by carboxyl (R-CO_2_H) and phosphate groups (R-PO_4_H_2_) of teichoic acids in Gram-positive bacteria, and phospholipids and lipopolysaccharides (LPS) in Gram-negative bacteria [39]. Bacterial S-layers further influence net surface charge depending on the presence or absence of S-layer glycol proteins with glycosylated long carbohydrate chains, and depending on the structural groups exposed within their lattice pores [40–42]. These bacterial surface structures are illustrated in Fig. 1. Extracellular polymeric substances (EPS), capsules, sheaths, slimes and biofilm matrices may further surround Gram-positive or Gram-negative bacteria. These are also usually associated with a net negative charge imparted by carboxyl and phosphate groups, which are free to interact with soluble cations [43].

The extent of the surface negative charge is governed by the deprotonation of functional groups with an increase in pH: carboxyl, phosphate, hydroxyl (R-OH) and sulphate (R-SO_4_) groups increase their negative charge, while amine (R-NH_2_) groups decrease their positive charge. For bacteria living in environments with neutral pH ranges, this means that surfaces tend to be negatively charged and have a high affinity for cationic species [44, 45]. Carboxyl groups in particular have been found to contribute strongly to the metal-binding capability. Studies on *Bacillus subtilis* used chemical modification of phosphate and carboxyl functional groups to demonstrate their importance in and relative contribution to metal ion binding [46, 47]. More recent studies of Gram-positive cell walls support this role, with half the binding of calcium and magnesium coming from polyphosphate groups of teichoic acids and half from carboxyl groups of peptidoglycan [48].

Teichoic and teichuronic acids, as well as LPS are natively stabilized by the presence of divalent cations, providing starting nucleation sites for mineral formation [43]. Surface cation binding sites are assumed to form the centre of crystal growth. Mineral precipitation occurs from nucleation of cations to previously adsorbed surface cations. The formation of these critical nuclei is stabilized by the surface functional groups through a reduction of tension between the bulk water phase and mineral nucleus [43]. Once bound, supersaturation is achieved by lowering the free energy necessary for precipitation, often with the help of metabolism-induced changes in pH. Consequently precipitation can then occur faster than in systems without bacteria [49]. For example, in the precipitation of the calcium-magnesium mineral dolomite, the dehydration of the magnesium ion and subsequent carbonation are the rate-limiting step of nucleation [50]. In the presence of carboxyl groups, [Mg(H_2_O)_6_]^2+^ binds and dehydrates to [Mg(H_2_O)_5_(R-COO)]^+^. This lowers the activation energy for subsequent carbonation and attachment of Ca^2+^ to form dolomite [CaMg(CO_3_)_2_] [50–52]. Thus, bacteria provide a mechanism of heterogeneous precipitation, with their surfaces acting as a nucleation catalyst and template, as well as increasing the saturation state through local attraction of cations.

Beyond the direct influence of bacterial surfaces, the microenvironment they create also plays a very important role in influencing ion saturation state. All submerged surfaces, such as those of micro-organisms, are surrounded by a thin-filmed water envelope called the hydrodynamic boundary layer [53]. Bacteria live at an extremely low Reynolds number, that is, the viscous forces of the environment dominate over their ability to move. As a consequence, these bacteria experience greater viscous drag and so struggle to escape their thin water envelope [53]. Within this surrounding water envelope, concentration gradients of ions can form where local concentrations are higher than in the bulk aqueous environment. Supersaturation will vary with ion concentration and so precipitation will be favoured within the cell-surface vicinity where the concentration is highest. The concentration gradient is the combined result of cell surfaces lowering thermodynamic activation energies, sequestering cations, as well as metabolic activity providing anions such as HCO_3_^-^, all of which occurs within the surrounding water layer [29, 54].

**Fig. 2.**
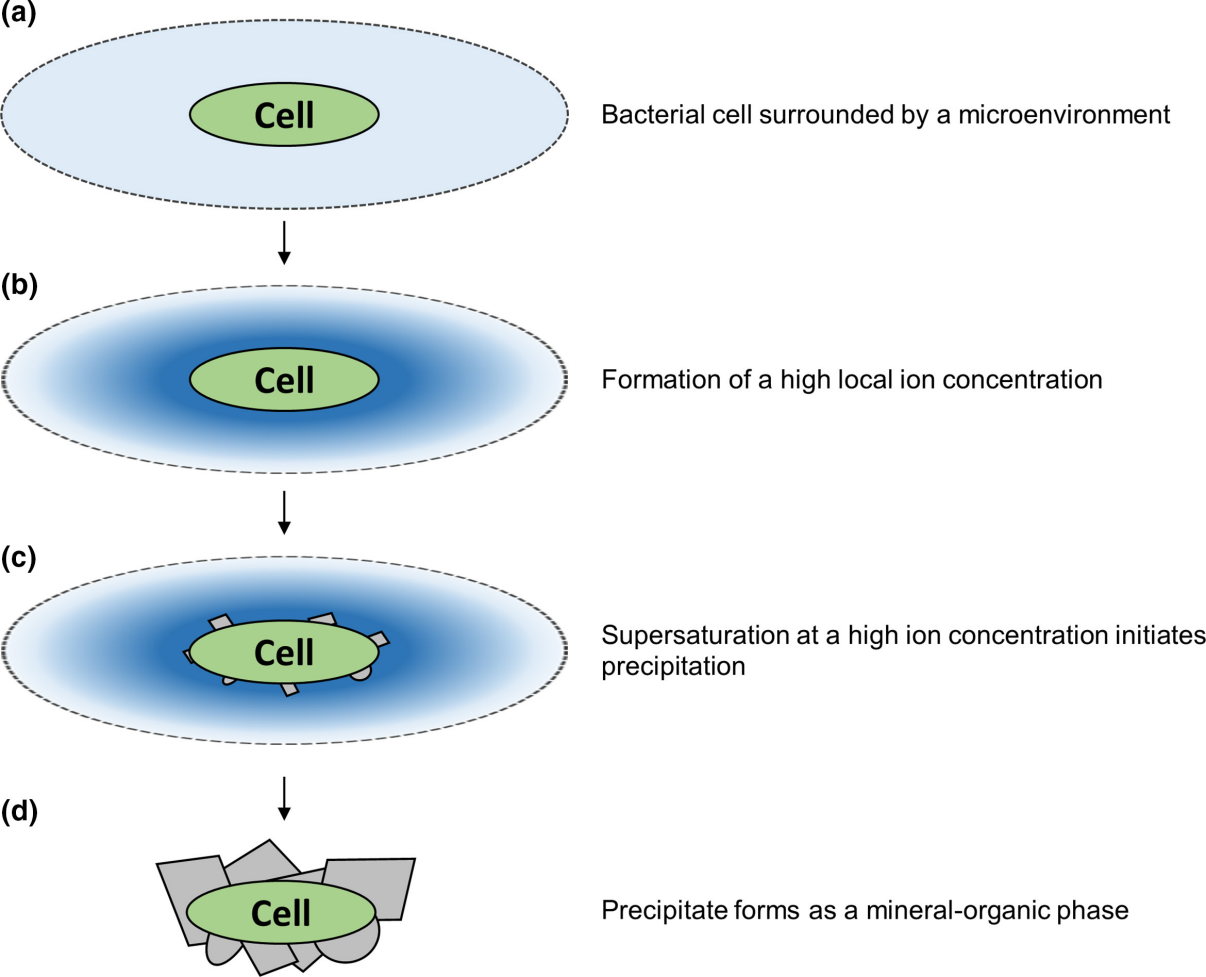
Mineral encasement of a bacterial cell. (a) Bacterial cell (green) surrounded by a microenvironment (light blue) created by an extremely low Reynolds number and/or sheaths, capsules, slimes, biofilm matrices or extracellular polymeric substances. (b) Accumulation, stabilization and slow diffusion of ions within the microenvironment close to the cell occurs from metabolism and cell-surface interactions creating a high local ion concentration (dark blue). (c) Within the cell-surface vicinity, at a high ion concentration, the equilibrium is shifted in favour of supersaturation and thus precipitation (grey shapes). (d) Onset of precipitation can lead to the breakdown of the microenvironment and, along with the degradation of some extracellular organic components, leaves behind a mineral-organic phase encasing the cell (grey shapes).

This principle can be extended further to other layers surrounding microbial surfaces such as biofilm matrices, slimes, sheaths, filaments, capsules and EPS secretions. These layers can create a microenvironment that favours supersaturation and thus precipitation via local changes in ion mobility, viscosity and nucleation kinetics (Fig. 2) [55]. For example, mineralization has been seen on bacterial sheaths and filaments [56, 57], slimes [58, 59], biofilms and EPS [60]. Some findings even showed that purified EPS alone could contribute to mineral precipitation, while other studies found that EPS production was not always associated with mineral precipitation [61–63]. This emphasizes the complexity of the process dependent on the bacterium, environment, mineral formed and underlying mechanism. In cyanobacterial systems, EPS has been shown to inhibit the precipitation in the bulk phase of the environment by trapping large amounts of divalent cations in its sugars, acidic residues and negatively charged functional groups. Only upon degradation of EPS and liberation of the cations does the saturation index increase, allowing for the precipitation of minerals [24, 64, 65].

Further to creating favourable conditions, the microenvironment is not subject to the same kinetics as the bulk environment and therefore also protects against inhibiting factors such as ion complexing and cation hydration [66]. Thus, bacterial surfaces and their microenvironments allow precipitation to occur even in unfavourable conditions such as acidic environments [67]. Over the course of precipitation and with the eventual degradation of some extracellular organic components, the microenvironment is broken down and leaves behind a mineral organic phase encasing the cell, illustrated in Fig. 2. Active mechanisms by which the bacteria can avoid or escape such encasement are discussed later in this article.

### Cell surface: polymorph ratio, crystal morphology, mineral type, and crystal size

In addition to providing nucleation sites and concentrating ions, surface structures can influence mineral polymorph ratio, crystal morphology and the type of minerals precipitated. Polymorphs have the same chemical structure but differ in their crystal structure [68]. Calcium carbonate mainly encompasses the polymorphs' calcite, vaterite and aragonite, and their ratios can be affected by cell-surface chemistry. For example, the presence of carboxylic groups, phosphonates, sulfonates and amino acids has been found to promote formation of vaterite [69]. The morphology of calcium carbonate crystals has been reported to be influenced through the presence of organic matter, e.g. by an increase in acidity of l-amino acids and xanthan content, where calcite crystals transitioned from rhombohedra to fibro-radial spherulites, and the monocrystals that make up the typical vaterite crystal spheres evolved from clustered short needles to clustered large hexagons [58].

The type of mineral precipitated is in part determined by the selective adsorption of metals to certain functional groups. Different metals were found to bind cell-surface components with different affinities. For example, it was reported that Mg^2+^ bound with a higher affinity than Ca^2+^ to cell walls of the Gram-positive *B. subtilis* [46, 47, 70] as well as to cell envelopes of the Gram-negative *Escherichia coli* [71]. The selective adsorption of calcium and strontium cations versus that of magnesium to pores within S-layers of *Synechococcus* sp. governed the preferred precipitation of the sulphate minerals gypsum and celestite [72].

### Cell surface or metabolism: are precipitating bacteria dead or alive?

Different observations have been reported regarding whether BIMP is strictly dependent on bacterial activity, specifically whether dead cells may be able to facilitate biomineralization. This leads to different interpretations of how important cell-surface structures are for the process of mineral precipitation. While materials science studies showed that precipitation can occur on functional group monolayers [73], absence of precipitation on dead cells suggests that the organic material is not simply a nucleation seed, but that metabolic activity also plays a key role [74, 75]. In contrast, other work found that minerals do form on dead cells and their debris [61, 76, 77]. This discrepancy may simply be a result of the differences between bacterial species, environmental conditions or methodologies used to prepare the dead cells, as this may affect structural properties [2]. Systematic studies would be required to determine how much of this variability in mineral precipitation on live versus dead cells is genuinely due to specific properties of the particular species investigated, or if other factors of experimental design or conditions are the main drivers of the outcome.

While an unequivocal answer to the question is currently lacking, considerations of the implications of BIMP in bacterial communities may shed some light. As described above, mineral precipitation on the cell surface leads to encasement of the cell (Fig. 2). Therefore, if only living cells precipitated minerals, the whole population could run the risk of entombment and death. To allow for the continued growth of a population, precipitation might therefore be assumed to occur only on dead cells and/or a restricted number of live cells [78]. On the other hand, mechanisms exist for active evasion of entombment by shedding encrusted S-layers [41], forming mineral sheaths/capsules [56], forming nanoglobules to act as decoy precipitation targets [79, 80], or even controlling surface functional group distribution to control precipitation occurrence [81].

The role of metabolism in evading entombment is also unclear. One observation has been that induction of a proton motive force by metabolic activity of live cells reduced the cell-wall metal-binding ability [82]. Metabolism as an active mechanism against entombment also has been proposed in cyanobacteria and suggested that dead cells could potentially be better at mineral precipitation because they retained more of their negative surface charge [83]. Zeta potential analysis was used to approximate the net surface charge of the bacteria by measuring the potential differences between the cell and fluid interface [84]. In these studies, metabolic activity was found to contribute to a more positive surface charge, likely regulated to attract anions for metabolism. On the other hand, dead cells retained a constant negative charge on their surface structures [84]. At a community level, i.e. a mixture of live and dead bacteria, one explanation for the ability of these bacteria to precipitate minerals on their surface may be as a result of cations binding to negatively charged surfaces of dead or inactive cells. Alternatively, a somewhat counterintuitive explanation might be the attraction of carbonate anions to metabolically active cells and letting these act as the seed for nucleation rather than the typical cations [84]. Evidence of changes in cell-surface charge between dead and live cells is still limited, and there is likely to be variability among bacterial species depending on their surface structures. Taking into account these observations, the more likely explanation is that most often both surface structure and bacterial metabolism are required as catalysts to modulate precipitation parameters by influencing saturation state and nucleation ability. Precipitation should occur under conditions of supersaturation, when cations attracted to the bacterial surface react with counter anions in the environment. Anion concentration is in turn environment dependent or may be supplemented by metabolism, suggesting both live and dead bacteria may be needed.

### Bacterial metabolism: pH and anions, including dissolved inorganic carbon (DIC)

Apart from the availability of nucleation sites, mineral precipitation also depends on (i) availability of anions, (ii) availability of cations and (iii) pH [85]. Bacterial metabolism plays an integral role in BIMP whereby it chemically alters the environment through the production of metabolites and by-products that influence the local pH and ion concentrations (e.g. carbonate, phosphate or metal cations). Modulation of these parameters ultimately affects supersaturation conditions and thus precipitation.

A key parameter to consider in mineral precipitation is the ion activity product (IAP), which for low-solubility minerals can be approximated as the product of the concentrations of the anion and cation composing the mineral, as exemplified for calcium carbonate in Equation 1. Supersaturation is achieved when the IAP of the mineral exceeds its solubility product constant (*K*_sp_), as defined in the saturation index (SI) (Equation 2). A system is considered supersaturated when SI>0 [13, 86, 87].

(Equation 1) $$IAP\;(CaCO_{3})=[Ca^{2+}]\times [CO_{3}^{2-}]$$

(Equation 2) $$SI=log\left(\frac{IAP}{K_{sp}}\right)$$

While *K*_sp_ is a constant for a given system, IAP depends on effective concentrations and can be influenced by environmental factors such as bacterial metabolism. The precise value of SI at which precipitation occurs spontaneously for a given system can vary, depending, for example, on the presence of organics that can promote precipitation or even inhibit it despite high saturation states [69, 86, 88–90]. In that regard, SI only predicts the point at which precipitation is thermodynamically favoured but not when it actually begins. In BIMP, the point at which precipitation is observed can in part depend on cell density and nucleation points [13, 60, 91–93], but it also critically depends on the effects of bacterial metabolism on IAP.

Metabolic activity is furthermore accompanied by changes in pH due to the production of various metabolic by-products. This in turn affects precipitation potential, with a higher pH directly contributing to the availability of anions through deprotonation and supersaturation. For example, in the case of mineral carbonates, the precipitation potential is dependent on both the pH and the carbonate anion concentration, known as the total dissolved inorganic carbon (DIC), which is the sum of the dissolved forms of CO_2_, HCO_3_^-^ and CO_3_^2-^. Moreover, the concentration of anions is directly related to the pH through the dissociation constants as seen in the carbonate equilibrium (Equation 3) [94]. At higher pH, the carbonate equilibrium is shifted to the right and carbonate species are deprotonated. As a result, more bicarbonate (HCO_3_^-^) and carbonate (CO_3_^2-^) ions are available for precipitation. Similarly, phosphate groups will be subject to changes in protonation state, depending on environmental pH (Equation 4). Sulphate groups will typically be present in their deprotonated state due to their low pKa values (usually below 2.5) (Equation 5), which will generally be exceeded by environmental pH [60]. Precipitation at low pH is possible in theory, but mostly applies to phosphate and sulphate-containing minerals where the anion component has a lower pKa. However, in practice, low pH often leads to dissolution of minerals.

(Equation 3) $$CO_{2}+H_{2}O\overset{pKa\;6.35}{↔}HCO_{3}^{-}+H^{+}\overset{pKa\;10.3}{↔}CO_{3}^{2-}+2H^{+}$$

(Equation 4) $$H_{3}PO_{4}\overset{pKa\;2.16}{↔}H_{2}PO_{4}^{-}+H^{+}\overset{pKa\;7.21}{↔}H_{2}PO_{4}^{2-}+H^{+}\overset{pKa\;12.32}{↔}H_{2}PO_{4}^{3-}+H^{+}$$

(Equation 5) $$H_{2}SO_{4}\overset{pKa-3.0}{↔}HSO_{4}^{-}+H^{+}\overset{pKa\;1.99}{↔}SO_{4}^{2-}+H^{+}$$

**Fig. 3.**
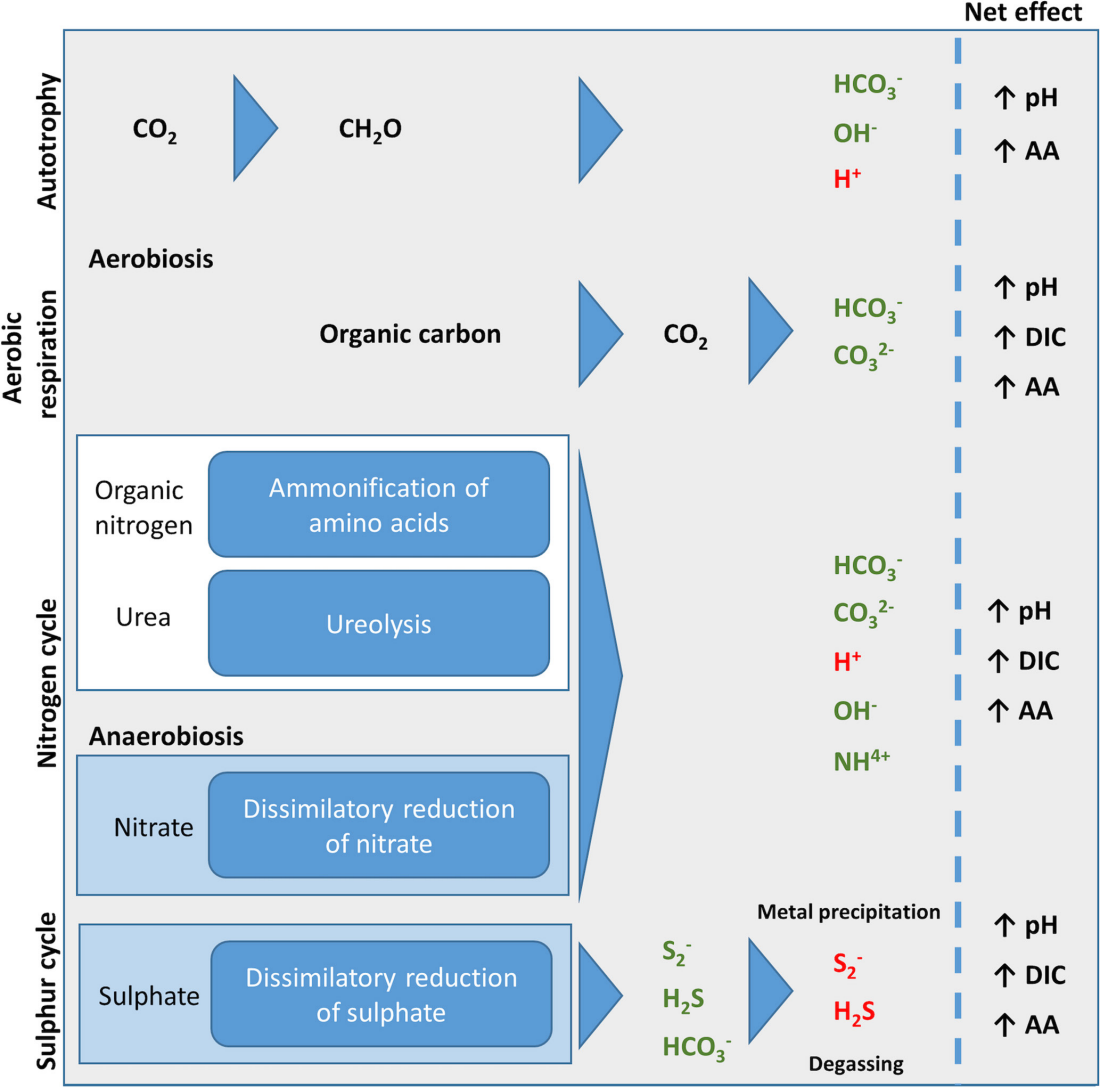
Metabolic pathways associated with bacteria-induced mineral precipitation. Various products of metabolism result in a net effect, shown on the right, that primes the environment for mineral precipitation. AA refers to anion availability, typically bicarbonate and carbonate. Products in green are increased and those in red are decreased as a result of metabolic activity. Adapted from [98].

Bacterial metabolism, through a modulation in pH and the production of anions such as phosphates, sulphates and carbonates, therefore has a direct influence on IAP and can increase the likelihood of anions and cations precipitating together as minerals [87]. Which anions are produced ultimately also depends on the availability of nutrients and metabolic capabilities of the specific bacteria present. For example, bacteria capable of reducing sulphate can produce sulphide ions that can directly precipitate as minerals, while bacteria that break down urea or amino acids increase the local pH, which in turn favours formation of carbonates for mineral precipitation (Fig. 3). For reasons of brevity, however, only the key contributing factors in terms of net ion production and pH effects created by different metabolic pathways contributing to mineral precipitation are discussed here. The specific physicochemical details of the various individual metabolic pathways that can induce mineral precipitation have been reviewed elsewhere [33, 95].

#### Autotrophic metabolic pathways

Autotrophic metabolic pathways such as non-methylotrophic methanogenesis or oxygenic and anoxygenic photosynthesis utilize CO_2_ to produce organic matter. This causes a depletion in CO_2_ that alters the bicarbonate equilibrium through a shift to the left (Equation 3), leading to removal of H^+^ as bicarbonate concentration increases, as well as dissociation of bicarbonate ions to CO_2_ and OH^-^. The resulting increase in pH favours precipitation under conditions of low DIC but high concentrations of suitable cations (Fig. 3) [24, 85].

#### Aerobic heterotrophic metabolism

Aerobic heterotrophic metabolism can cause local increases in anion concentration and pH. As mentioned above, aerobic heterotrophs break down organic carbon to produce CO_2_ that partially converts to carbonate and bicarbonate and increases DIC and pH in the bulk phase [64, 96].

#### Nitrogen cycle

Dissimilatory reduction of nitrate under anoxic conditions and deamination of amino acids for their catabolic use both lead to production of ammonium and hydroxide ions and consumption of H^+^ ions. This causes an increase in pH and thus shifts dissociation equilibria of anions that are relevant for mineral formation (Fig. 3) [96]. The role of ureolysis in mineral precipitation is explained below.

#### Sulphur cycle

Dissimilatory reduction of sulphate, carried out in anoxic conditions by sulphate-reducing bacteria, results in the production of carbonate, bicarbonate and hydrogen sulphide (H_2_S) (Fig. 3). Whether this leads to biomineralization depends on the fate of the H_2_S produced. Excreted sulphide can lead to authigenic precipitation in the bulk phase by directly reacting with metal cations in the environment to precipitate sulphide minerals [97]. Alternatively, loss of H_2_S can occur through degassing or consumption by anoxygenic sulphide phototrophic bacteria that oxidize H_2_S to elemental sulphur and form intra- or -extracellular deposits. The removal of H_2_S increases the pH and thus favours precipitation (Fig. 3) [98]. On the other hand, autotrophic sulphide-oxidizing aerobic bacteria use H_2_S (and other reduced sulphur compounds, S^0^ and S_2_O_3_^2-^) to produce sulphate ions that form sulphuric acid, decreasing the pH and dissolving precipitates [64, 98]. The balance between precipitation and dissolution therefore will be dependent on environmental conditions such as oxygen availability, light and pH, which serve to decouple the different metabolic processes in time and space and establish local conditions where net precipitation can occur [99].

### Single enzyme-mediated reactions

Aside from broader metabolic pathways, specific enzymes can also contribute to precipitation. Acid phosphatases liberate phosphoryl groups, thus accelerating formation of phosphate mineral species, and strains overproducing this enzyme were shown to precipitate uranium phosphate species [34, 100–102]. However, not all bacteria with phosphatase activity can precipitate minerals, lending weight to the idea that specific cell-surface structures are likely required to provide nucleation sites for precipitation [103].

Carbonic anhydrase, catalysing the interconversion of CO_2_ to HCO_3_^-^ and H^+^, has been suggested as a key enzyme in precipitation due to its effect on local HCO_3_^-^ concentration. The presence of extracellular carbonic anhydrase was found to govern the location of crystal precipitates in biofilms of *Alcanivorax borkumensis* [96]. Indeed, carbonate precipitation was restricted to areas with high extracellular concentration and activity of carbonic anhydrase.

Ureolysis as part of the nitrogen cycle is also an enzymatically driven process. This enzymatic activity may potentially be strong enough to increase supersaturation to such high levels that precipitation can occur without the need for nucleation sites provided by bacterial cell surfaces [104]. Indeed, it was observed that some strongly ureolytic bacteria could induce calcite precipitation at a considerable distance to the bacterial colony [32].

While the processes described in this review, i.e. the complex interplay between physical properties of bacterial cells and their metabolic activity, explain why in BIMP one is more likely to encounter heterogenous precipitation, strongly ureolytic bacteria may, in fact, be an exception and capable of driving homogenous nucleation.

### Cell metabolism: provision of cations

Apart from the generation of anion species needed for precipitation, cation availability can also be influenced through metabolic activity. As defined within the IAP, the concentration of the metal cation is also important for the precipitation of a mineral species (Equation 1). Bacteria, often via enzymatic activities, may reduce a mineral compound to produce divalent cations that can then react with anions to precipitate as a different mineral [105, 106]. Some bacteria utilize metal ions as terminal electron acceptors in microaerobic or anaerobic conditions to produce cations, for example Fe^2+^ through reduction of oxidized iron (Fe^3+^), usually from dissolution of other iron oxides, as reviewed in detail elsewhere [106–108]. The resulting Fe^2+^ can subsequently interact with various anions to form a variety of iron minerals (Table 1). Many iron-reducing bacteria are also capable of reduction of manganese (Mn^4+^ to Mn^2+^), providing Mn^2+^ cations for mineral formation [59]. Metal oxidation can also occur under anoxic conditions through the activity of some phototrophic bacteria and some nitrate-respiring bacteria [59].

Local cation concentration can also fluctuate due to active bacterial processes such as intracellular metal ion homeostasis via ionic pumps and channels. In high-calcium environments, such as calcareous caves and limestone soils, the need to maintain a low intracellular calcium concentration is essential to ensure bacterial survival and growth [109]. Microbes can achieve this through active efflux of intracellular calcium by ATP-dependent antiporters, increasing the local calcium availability and pH near the cell surface and thus contributing to precipitation [85, 110]. Thus, active calcium efflux could be seen to influence biomineralization in two ways: it ensures bacterial growth to provide nucleation sites while simultaneously increasing the local cation concentration. Indeed, active processes of ion excretion may precede the passive precipitation discussed previously and allow microbes to act as nuclei for subsequent crystal growth [98].

### Prospects

In exploring the underlying processes enabling BIMP, a lot of benefit has been gained from research across multiple disciplines investigating the different aspects of organic-mineral interphases. While there are mechanistic differences in the way bacteria induce mineralization dependent on their surface architecture and metabolism, understanding the contributing components is important for biotechnological application. An additional layer of complexity is introduced when considering that bacteria do not occur in isolation, and that metabolic processes of one group of organisms are often interdependent with the activities of other groups. Indeed, in nature precipitation results from the activities of mixed populations, which often grow as biofilms rather than planktonic cells [24, 98]. This could possibly be exploited in utilizing communities and biofilm growth of micro-organisms to maximize precipitation potential. BIMP has seen increased applications in civil engineering and biotechnology over recent years, as extensively reviewed elsewhere [10, 12–14, 87].

In brief, mineral precipitation mainly has two different roles in these technologies. For applications that include soil consolidation, heritage conservation and self-healing concrete, precipitated minerals and embedded cells and organic components become the ‘glue’ that binds and/or seals the surrounding matrix. For applications of bioremediation such as of toxic heavy metals or radionuclides or in carbon dioxide sequestration, the elements in question are directly precipitated or co-precipitated, rendering them bio-unavailable [13, 14]. Fundamental mechanistic insight will therefore be important in making more informed decisions in choosing the appropriate bacteria for a specific application in terms of strain characteristics and minerals precipitated. This could allow for selective mineral precipitation, dependent on preferential surface binding and metabolic anion production of the chosen bacterium. Additionally, one could even modulate the speed of precipitation through the choice of metabolic capability, depending on application need. In the future, detailed mechanistic insights may inform rational directed evolution or genetic engineering approaches for application-driven strain development. The complexity of BIMP and its dependency on precise bacterial properties may therefore even be viewed as a benefit. Nature may reveal a useful and versatile toolbox of different bacteria, supplemented by systematic strain engineering to meet future needs for sustainable BIMP technologies.
